# Multivariable model integrating PHI and mpMRI for detecting csPCa in biopsy‐naïve men

**DOI:** 10.1002/bco2.70101

**Published:** 2025-12-02

**Authors:** Mario Dominguez Esteban, Ester Fernandez Guzman, Enrique Ramos Barselo, Ernesto Herrero Blanco, Sergio Zubillaga Guerrero, Roberto Ballestero Diego, Alejandro Fernandez Florez, Jose Javier Gomez Roman, Jaime Garcia Herrero, Marina Sanchez Gil, Guillermo Velilla Diez, Felix Campos Juanatey, Maria Teresa Garcia Unzueta, Jose Luis Gutierrez Baños

**Affiliations:** ^1^ Department of Urology University Hospital Marques de Vadecilla‐IDIVAL Santander Spain; ^2^ Department of Radiology University Hospital Marques de Vadecilla Santander Spain; ^3^ Department of Pathology University Hospital Marques de Vadecilla Santander Spain; ^4^ Department of Urology University Hospital Marques de Vadecilla Santander Spain; ^5^ Department of Biochemistry and Clinical Análisis University Hospital Marques de Vadecilla‐ IDIVAL Santander Spain

**Keywords:** clinically significant, prostate cancer, mpMRI, multivariable model, nomogram, PHI, PIRADS, prostate cancer, prostate health index, risk stratification

## Abstract

**Background:**

The integration of blood‐based biomarkers and multiparametric magnetic resonance imaging (mpMRI) has been proposed to improve prostate cancer (PCa) diagnosis. However, few validated models combine both tools to support risk‐adapted clinical decision‐making.

**Objective:**

The study's aim is to evaluate and internally validate a multivariable model integrating clinical, analytical and imaging parameters—including the Prostate Health Index (PHI) and mpMRI—for predicting clinically significant prostate cancer (csPCa) in biopsy‐naïve men.

**Design, setting and participants:**

This prospective observational study included 183 biopsy‐naïve men aged 50–75 years with PSA levels of 4–10 ng/mL and/or abnormal digital rectal examination. All patients underwent PHI testing, and 47.5% received prebiopsy mpMRI. All underwent systematic biopsy; targeted cognitive fusion biopsy was performed for PIRADS ≥ 3 lesions.

**Outcome measurements and statistical analysis:**

A multivariable logistic regression model was constructed using PHI, PSA density, PSA free/total ratio, PIRADS score and age. The model was internally validated with bootstrap resampling and converted into a clinical nomogram. Diagnostic accuracy (AUC, sensitivity, specificity, NPV and PPV) was assessed and compared with simplified strategies using PHI or PIRADS alone, as well as a sequential approach (PHI → PIRADS).

**Results and limitations:**

The model achieved an AUC of 0.841 (95% CI 0.76–0.91), with 100% sensitivity and 66.7% specificity for csPCa in the mpMRI cohort at the optimal 17% risk threshold (65.5 points). It safely avoided 49.4% of biopsies without missing any csPCa cases. Simpler strategies using PHI or PIRADS alone showed lower efficiency, particularly in balancing sensitivity and biopsy reduction. As an additional analysis, the PHI–mpMRI nomogram by Siddiqui et al. (2023) was externally validated in our cohort, confirming robust diagnostic accuracy (AUC 0.89, 95% CI 0.82–0.95). Limitations include the modest size of the mpMRI cohort and the historical nature of recruitment (2014–2018), although PHI and mpMRI remain standard in contemporary practice.

**Conclusions:**

This model accurately predicts csPCa and outperforms individual tools such as PHI or PIRADS alone. Its application may improve diagnostic efficiency and reduce unnecessary procedures.

**Patient summary:**

A model combining a blood test (PHI) and MRI can help avoid unnecessary prostate biopsies while reliably detecting aggressive cancers.

## 1 INTRODUCTION

Prostate cancer (PCa) is the most frequently diagnosed malignancy in men and remains one of the leading causes of cancer‐related death worldwide.^1^ According to GLOBOCAN, over 1.4 million new cases were diagnosed in 2020, and this figure is projected to surpass 1.7 million by 2030.^1^ In Europe—and particularly in countries like Spain—its incidence continues to rise, driven by population aging and the widespread use of prostate‐specific antigen (PSA) testing.^2^, ^3^

PSA‐based screening has been shown to reduce PCa‐specific mortality, as evidenced by the long‐term outcomes of the ERSPC study with 16 years of follow‐up.^4^ However, this benefit has been accompanied by substantial overdiagnosis and overtreatment, with significant clinical, psychological and economic implications.^5^, ^6^ In fact, studies on conservative management of localized PCa have shown that many low‐risk tumours follow an indolent course, with a low 20‐year cancer‐specific mortality risk in such cases.^5^

To address the limitations of PSA, several complementary tools have been developed to improve patient selection for prostate biopsy. Among these, the prostate health index (PHI) has demonstrated greater diagnostic specificity, enabling better discrimination of clinically significant cancers versus indolent disease.^7^, ^8^ More recently, prospective studies in large cohorts, such as that of Chiu et al. conducted in Hong Kong, have confirmed its utility in real‐world clinical settings.^9^

Concurrently, multiparametric magnetic resonance imaging (mpMRI) has become a key tool for initial diagnosis and staging of PCa. Landmark studies such as PRECISION, MRI‐FIRST and 4M have shown that mpMRI followed by targeted biopsy improves detection of clinically significant cancer while reducing the diagnosis of low‐risk tumours.^10^, ^11^, ^12^ Despite its advantages, mpMRI has certain limitations related to availability, cost and inter‐reader variability, especially in settings with less radiological expertise.^13^, ^14^, ^15^

Although several studies have evaluated the combined performance of biomarkers like PHI with imaging tools such as mpMRI,^16^, ^17^, ^18^, ^19^ a clear and scalable clinical pathway for their joint implementation remains lacking. Models based exclusively on mpMRI, such as that proposed in the Göteborg–2 study, have raised concerns regarding cost‐effectiveness.

Recently, Patel et al.^20^ reviewed the landscape of predictive models based on mpMRI, highlighting that although several risk calculators exist, very few incorporate advanced serum biomarkers such as PHI. Among the exceptions, Siddiqui et al.^21^ developed a nomogram integrating PHI, PSA density, age, and PIRADS, which achieved excellent diagnostic accuracy in a large contemporary cohort. However, this tool has not yet been externally validated in European prospective series. This underscores the clinical relevance of evaluating integrative models that combine imaging with blood‐based biomarkers in biopsy‐naïve patients.

In this study, we aimed to assess the diagnostic value of PHI and mpMRI—independently and in combination—through a prospectively collected cohort of biopsy‐naïve men. Our primary objective was to develop and internally validate a multivariable predictive model for clinically significant PCa, integrating analytical, imaging, and clinical parameters. As a secondary aim, we also performed the first prospective European external validation of the recently proposed PHI–mpMRI nomogram by Siddiqui et al.^21^

## 2 MATERIAL AND METHODS

This was a prospective, observational study involving a cohort of patients evaluated for suspected PCa between 2014 and 2018. Eligible participants were men aged 50 to 75 years (or over 40 with a family history of PCa) and an estimated life expectancy greater than 10 years. Inclusion criteria included PSA levels between 4 and 10 ng/mL and/or a suspicious digital rectal examination, with no prior prostate biopsy. Exclusion criteria were active urinary tract infection, bladder stones, recent catheterization, ongoing hormonal therapy or use of 5α‐reductase inhibitors, severe renal impairment (MDRD < 20), significant protein alterations, haemophilia, recent blood transfusion or absolute contraindications to magnetic resonance imaging.

All patients underwent blinded PHI testing using validated kits (Beckman Coulter®), and the results were concealed from the clinical team to avoid influencing diagnostic decisions. Independently, approximately 50% of the patients underwent prebiopsy mpMRI, without PHI results affecting the indication for imaging. Images were interpreted by experienced radiologists using the current version of the PI‐RADS system at the time of the study.

All patients subsequently underwent transrectal ultrasound‐guided prostate biopsy. For those with mpMRI available, a systematic biopsy (≥ 12 cores) was performed along with cognitive‐targeted biopsy of lesions scored PIRADS 3, 4 or 5. In patients without mpMRI, a standard systematic biopsy protocol was followed.

Clinically significant PCa was defined as tumours with a Gleason score ≥ 3 + 4. Tumours classified as very low risk included those with a Gleason score of 3 + 3 in ≤2 cores, involving < 50% and/or < 6 mm of affected tissue per core.

Clinical, demographic and pathological characteristics were collected prospectively. A comparative analysis between patients with and without mpMRI revealed no statistically significant differences in key variables such as age, PSA, PSA density, PSA f/t, PHI, or the prevalence of clinically significant PCa, supporting the validity of extrapolating diagnostic models to the full cohort.

### 2.1 Model development and validation

A multivariable logistic regression model was constructed to predict the presence of clinically significant PCa. The model incorporated the following predictors: PHI, PSA density, PSA free/total ratio, PIRADS score and age. Model performance was evaluated using the area under the receiver operating characteristic curve (AUC), sensitivity, specificity, positive and negative predictive values, and odds ratios (OR) with 95% confidence intervals (CIs). The final model was converted into a clinical nomogram.

The model was first trained using the subset of patients with complete data for all predictors, including mpMRI (*n* = 81). This step focused exclusively on parameter estimation, ensuring consistency and minimizing bias from missing data—particularly PIRADS score, which was only available in patients who underwent imaging.

In a second phase, the trained model was applied to the entire study cohort (*n* = 184) to simulate its potential impact at a population level. For patients without mpMRI, PIRADS values were extrapolated to mirror the distribution observed in the imaged subgroup. Importantly, this step was used exclusively for exploratory simulations to estimate the potential clinical impact and was not included in model training or internal validation. In sensitivity analyses, men without MRI were alternatively categorized as a separate group (‘No mpMRI’), alongside PIRADS 1–2, 3, 4 and 5, to avoid any risk of overfitting. The optimal decision threshold was identified using the Youden index, balancing sensitivity and specificity.

In the final phase, the model and its decision threshold were applied back to the original 81‐patient cohort with mpMRI and complete data to assess its diagnostic utility under real‐world conditions. This three‐step approach—model development, simulated extrapolation, and internal validation—provided a rigorous framework for evaluating the robustness and scalability of the proposed strategy.

All statistical analyses and multivariable model development were conducted using Python (version 3.10), employing standard libraries for data processing (pandas, numpy), statistical analysis (scikit‐learn, statsmodels) and data visualization (matplotlib, seaborn).

## 3 RESULTS

A total of 183 patients with PSA levels between 4 and 10 ng/mL underwent transrectal ultrasound‐guided prostate biopsy. All patients had PHI values available, and 87 of them (47.5%) underwent multiparametric MRI (mpMRI) prior to biopsy. The clinical, demographic and pathological characteristics of the entire cohort are presented in Tables S1 and S2 (Appendix).

A comparative analysis between the groups with and without mpMRI revealed no statistically significant differences (see Table S3, appendix). This supported the homogeneity of the cohort and the validity of extrapolating the model's application to broader clinical settings.

PCa was diagnosed in 91 patients (49.7%), of whom 46 (25.1%) had clinically significant tumors (defined as Gleason score ≥ 3 + 4).

### 3.1 Diagnostic performance of PHI and PIRADS

As individual predictors of clinically significant PCa, both PHI and PIRADS demonstrated strong diagnostic performance:

- The ROC curve for PHI showed an AUC of 0.83. The optimal cutoff determined by the Youden index was 35.0, with a sensitivity of 85%, specificity of 67% and negative predictive value (NPV) of 92%.
- Among the 87 patients who underwent mpMRI, PIRADS showed an AUC of 0.84 for detecting clinically significant PCa. A PIRADS score ≥ 3 yielded a sensitivity of 89%, specificity of 63% and NPV of *91%*.

### 3.2 Comparative performance of simplified diagnostic strategies

To further contextualize the performance of the multivariable model, we assessed the diagnostic utility of PHI and PIRADS as individual tools, as well as in a sequential approach (PHI → PIRADS). The results are summarized in Table 1.

**TABLE 1:** Comparative performance of different diagnostic strategies for detecting clinically significant prostate cancer (csPCa).

Comparative Table of diagnostic strategies							
#	Strategy	*N* (patients evaluated)	Biopsies avoided (*n*)	Biopsies avoided (%)	csPCa not detected (n)	Total csPCa cases	Sensitivity (%)
1	PHI ≥ 35 (entire cohort)	184	66	35.9	4	47	91.5
2	PI‐RADS ≥ 3 (MRI only)	87	24	27.6	1	26	96.2
3	PHI ≥ 35 → PI‐RADS ≥ 3 (MRI only)	87	37	42.5	2	26	92.3

*Note*: The number and percentage of biopsies avoided, csPCa cases missed and sensitivity are shown for each strategy.

When applying PHI alone with a threshold of 35 to the entire cohort (*n* = 184), 35.9% of biopsies would have been avoided, with a sensitivity of 91.5% for clinically significant prostate cancer (csPCa). Using PIRADS alone (cutoff ≥ 3) among patients with available mpMRI (*n* = 87), 27.6% of biopsies would be avoided, with a sensitivity of 96.2%. Finally, a sequential strategy whereby only patients with PHI ≥ 35 underwent MRI and were subsequently biopsied if PIRADS ≥ 3 would have avoided 42.5% of biopsies while maintaining a sensitivity of 92.3%.

These results support the value of combining both serum and imaging markers to optimize diagnostic efficiency while minimizing the risk of missing significant cancers.

### 3.3 Multivariable model and nomogram

A multivariable logistic regression model was developed using the subset of 81 patients with complete data, including mpMRI. The predictors included the following: PHI, PSA density, PSA free/total ratio (PSA l/t), PIRADS score and age. The model demonstrated excellent discriminative capacity with an area under the ROC curve (AUC) of 0.841. Table 2 summarizes the model coefficients and odds ratios.

**TABLE 2:** Multivariable logistic regression model for the prediction of clinically significant prostate cancer.

Multivariable logistic regression model for csPCa prediction			
Variable	*β* coefficient	OR	95% CI
Intercept	−8.685	—	—
PHI	0.053	1.054	1.027–1.082
PSA density	0.015	1.015	0.966–1.065
PSA l/t ratio	−1.882	0.152	0.002–13.332
PIRADS score	0.663	1.941	1.21–3.112
Age	0.041	1.042	0.981–1.108

*Note*: Reported are *β* coefficients, odds ratios (OR), and 95% confidence intervals (CI) for each variable included in the final model.

**FIGURE 1:**
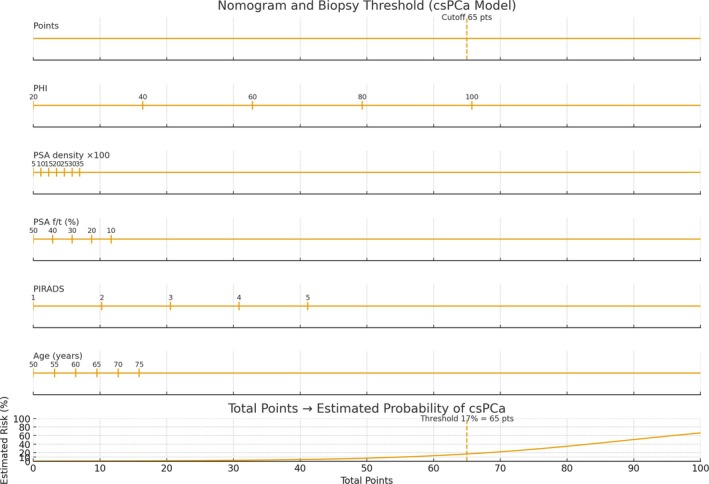
Nomogram for predicting the probability of clinically significant prostate cancer (csPCa, Gleason score ≥ 3 + 4). Each variable (PHI, PSA density, PSA l/t ratio, PIRADS score and age) contributes a number of points on the upper scale. The total score corresponds to an estimated probability of harbouring csPCa. *the x‐axis represents the total score calculated from the nomogram; the y‐axis indicates the estimated probability (%) of clinically significant PCa. The red dashed line marks the optimal 17% threshold (cutoff of 65.5 points) determined using the Youden index.

**FIGURE 2:**
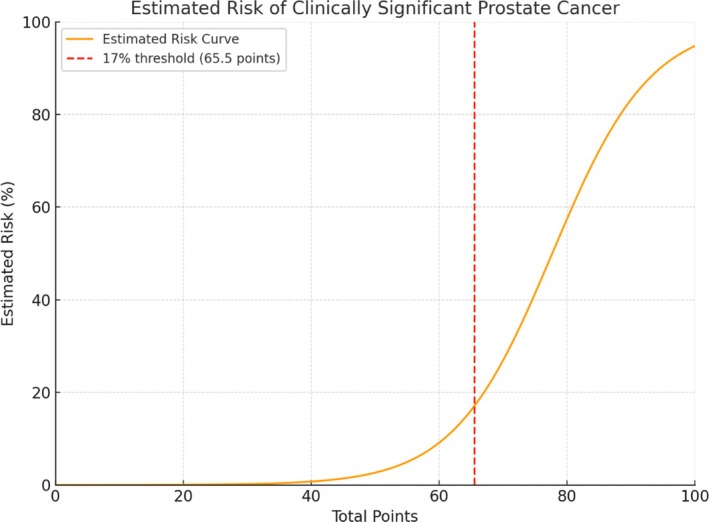
Calibration curve of the nomogram for predicting clinically significant prostate cancer (csPCa). The *x*‐axis shows the total points derived from the nomogram, and the *y*‐axis represents the estimated probability of csPCa. The orange line depicts the estimated risk curve, while the red dashed line indicates the optimal threshold of 17% (corresponding to 65.5 points) as determined by the Youden index.

The final model was transformed into a clinical nomogram (see Figures 1 and 2) for individualized risk estimation. A 17% probability threshold was defined as optimal using the Youden index, balancing sensitivity and specificity.

### 3.4 Real‐world application of the model (mpMRI cohort, *n* = 81)

Once finalized and calibrated using the optimal 17% threshold derived from diagnostic simulations, the multivariable model was retrospectively applied to the real‐world subgroup of 81 patients with complete clinical, analytical and imaging data.

When applied to this cohort, the model demonstrated strong discriminative capacity, with an area under the ROC curve (AUC) of 0.820. For the purposes of internal validation, a bootstrap analysis with 1000 iterations was performed, yielding a mean AUC of 0.772 (95% CI: 0.664–0.922). This confirms the model's robustness despite the moderate sample size.

**FIGURE 3:**
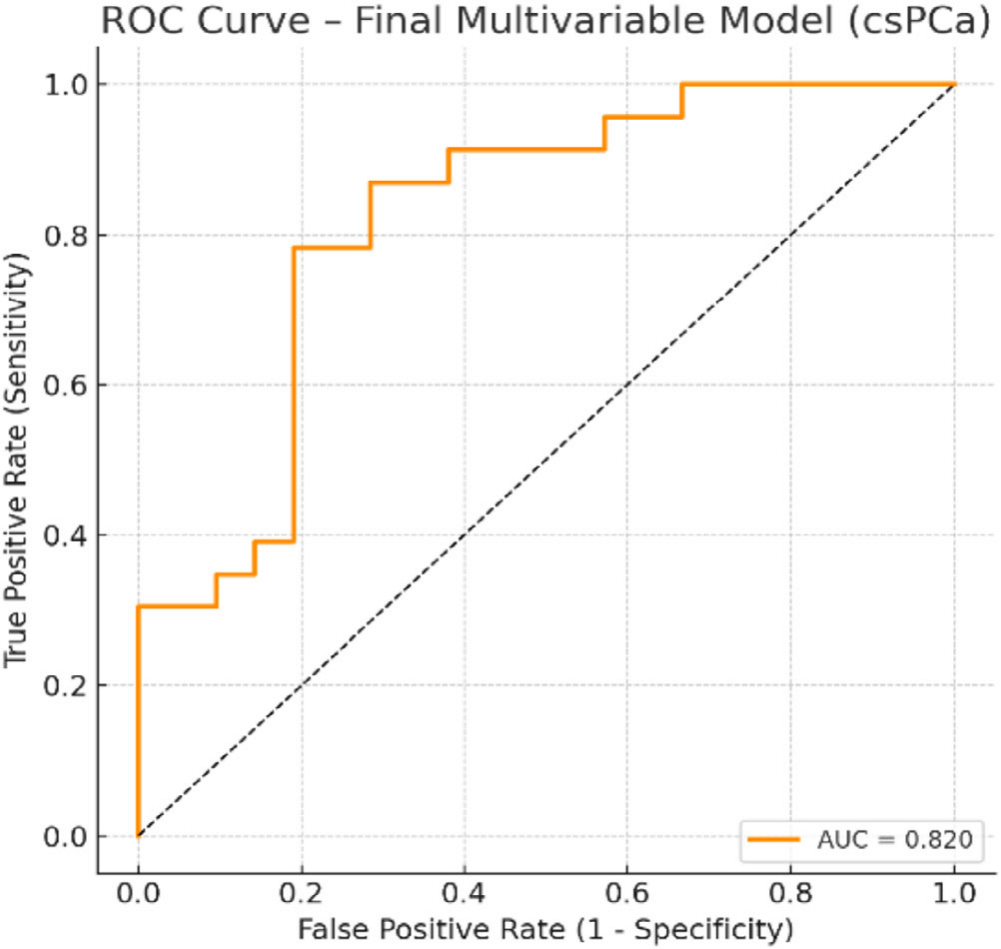
Receiver operating characteristic (ROC) curve of the final multivariable logistic regression model for detecting clinically significant prostate cancer (csPCa). The model achieved an AUC of 0.820. Internal validation with 1000 bootstrap iterations yielded a mean AUC of 0.772 (95% CI: 0.664–0.922).

This performance is graphically represented in Figure 3, where the ROC curve illustrates the model's ability to distinguish between patients with and without clinically significant disease. In Table 3 are described the odds ratios (OR) with 95% CIs and relative risk change for each predictor in the final logistic regression model developed using the mpMRI cohort (*n* = 81). According to the model, 41 patients (50.6%) would have been recommended for prostate biopsy, while 40 (49.4%) would have been spared the procedure. Among those classified as biopsy candidates by the model, 23 patients were diagnosed with csPCa, including 13 with Gleason 3 + 4 and 10 with Gleason ≥ 4 + 3. In contrast, none of the patients who would have avoided biopsy were found to harbour csPCa. This group included 33 patients with negative biopsy results and 7 patients with Gleason 3 + 3, consistent with very low or low‐risk profiles.

**TABLE 3:** Odds ratios (OR) with 95% confidence intervals (CI) and percentage change in risk for each predictor in the final logistic regression model, developed using the mpMRI cohort (*n* = 81).

Variable	OR	95% CI lower	95% CI upper	% change in risk
PHI	2.92	2.26	3.80	192.6
PSA density	0.96	0.74	1.25	−3.8
PSA l/t	0.44	0.35	0.55	−56.1
Age	1.02	0.82	1.28	2.1
PIRADS	3.94	3.10	5.01	293.9

At the 17% threshold, the model achieved the following:

- Sensitivity: 100%
- Specificity: 66.7%
- Positive Predictive Value (PPV): 56.1%
- Negative Predictive Value (NPV): 100%

These findings reinforce the model's clinical utility in safely reducing unnecessary prostate biopsies while maintaining high diagnostic safety.

### 3.5 External validation of the Siddiqui nomogram

We additionally evaluated the performance of the nomogram proposed by Siddiqui et al.,^21^ which integrates PHI, PSA density, age, and PIRADS. A total of 86 patients from our mpMRI subcohort were eligible, as this model does not require PSA free/total ratio, explaining the difference compared to the 81 patients used for our own multivariable model. Model discrimination, calibration and clinical utility were assessed as in the original publication. In our mpMRI subcohort (*n* = 86), this model achieved an AUC of 0.89 (95% CI: 0.82–0.95), with a calibration slope of 1.16 and Brier score of 0.12. Using the recommended 20% risk threshold, sensitivity was 96.2%, specificity 68.3%, PPV 56.8% and NPV 97.6%, allowing 48.8% of biopsies to be avoided. These figures are consistent with the original publication and represent the first external validation of this nomogram in a European prospective cohort.

## 4 DISCUSSION

The results of this study confirm that both the PHI and mpMRI demonstrate high diagnostic performance in identifying csPCa, consistent with previously published evidence.^9^, ^10^, ^11^, ^12^, ^17^ Beyond their individual utility, this work presents a validated multivariable model that integrates biomarkers and imaging parameters to improve clinical decision‐making in biopsy‐naïve patients.

The model, which includes PHI, PSA density, PSA free/total ratio (PSAlt3), PIRADS score and age, achieved an AUC of 0.841 and demonstrated excellent clinical performance in the real‐world cohort with mpMRI. When applied with the optimal risk threshold (17%) determined by simulation, it correctly identified all csPCa cases while avoiding biopsy in nearly half of the patients. This level of performance notably exceeds that of the individual variables alone.

The discriminative capacity of PHI observed in this cohort (AUC: 0.83) supports its value in risk stratification and in reducing unnecessary procedures, as previously reported in studies such as those by Chiu et al. and in the PRIM Study.^9^, ^17^ A PHI cutoff > 35 achieved high sensitivity and specificity, reinforcing its role as a reliable biomarker for prebiopsy decision‐making.^7^, ^8^, ^19^

Likewise, mpMRI showed robust individual performance (AUC: 0.84), aligning with findings from pivotal trials such as PRECISION and MRI‐FIRST, which have established mpMRI as the imaging modality of choice in the prebiopsy setting.^11^, ^12^ In our model, the addition of PIRADS significantly enhanced predictive accuracy, underscoring the synergistic potential of combining PHI and mpMRI—as suggested by recent combinatorial analyses.^22^

A key strength of this study lies in the availability of biopsy results for all patients, allowing objective evaluation of diagnostic accuracy. Importantly, PHI results were blinded to the clinical team, ensuring that this biomarker did not influence biopsy decisions. In contrast, mpMRI—when available—was used to guide cognitive fusion biopsies in addition to systematic sampling, which reflects routine clinical practice and may explain the higher number of cores obtained in this subgroup. The model's internal validation in a real‐world mpMRI‐based cohort, with 100% sensitivity for csPCa and a high NPV, supports its applicability in clinical settings. The additional validation of the Siddiqui et al. nomogram^21^ in our cohort further supports the clinical utility of combining PHI and mpMRI. While their model, derived from a large contemporary dataset, achieved excellent discrimination, our multivariable approach yielded comparable results with slightly higher NPV and biopsy‐sparing efficiency. As the intercept of the Siddiqui model was not available, our validation was limited to discrimination and calibration metrics rather than absolute risk prediction.

Several limitations should be acknowledged. First, our cohort size was relatively small, and recruitment ended in 2018. Although mpMRI has become increasingly used in recent years, our study represents a prospective series in which PHI was systematically assessed and all men underwent biopsy, minimizing selection bias. Second, the extrapolation of PIRADS values in non‐imaged patients was performed exclusively for exploratory simulations and not for model training or validation. To further address this limitation, we conducted a sensitivity analysis categorizing patients without imaging as a separate group (‘No mpMRI’). Third, inter‐reader variation in mpMRI interpretation and biopsy targeting—both influenced by local expertise—may limit generalizability. Finally, our model has not yet undergone external validation, which will be required in larger, contemporary multicenter cohorts before clinical implementation.

This study reinforces the value of integrated predictive tools to guide prostate biopsy decisions, especially in scenarios where multiple diagnostic elements must be considered. The nomogram derived from the model provides a user‐friendly interface for clinical application and may support shared decision‐making with patients.

This study reinforces the value of integrated predictive tools to guide prostate biopsy decisions, especially in scenarios where multiple diagnostic elements must be considered. The nomogram derived from our model provides a user‐friendly interface for clinical application and may support shared decision‐making with patients. In parallel, the external validation of the Siddiqui et al. nomogram^21^ in our cohort further confirms the robustness of PHI–mpMRI–based strategies across populations. Future studies should explore its integration with emerging tools, including SelectMDx, 4Kscore,^16^ or micro‐ultrasound imaging,^18^ as well as its adaptation into digital clinical decision support systems, in line with initiatives such as the ReIMAGINE study.^23^

## 5 CONCLUSIONS

This study demonstrates that the integration of clinical, analytical (PHI, PSA density, PSA l/t) and imaging variables (PIRADS score) into a multivariable model enables accurate prediction of csPCa in biopsy‐naïve patients. The model was internally validated in a real‐world cohort of patients with mpMRI and demonstrated excellent diagnostic performance, with 100% sensitivity and 66.7% specificity for clinically significant disease at a 17% risk threshold. The negative predictive value reached 100%, supporting its potential to safely reduce unnecessary biopsies while maintaining high diagnostic certainty. Additional analyses showed that simplified strategies based on PHI or PIRADS alone—or in sequential combination—were less effective in balancing diagnostic yield and biopsy reduction. Furthermore, the concordant results obtained with the Siddiqui nomogram emphasize the robustness of PHI–mpMRI–based approaches across populations. These findings support the implementation of integrated predictive strategies in urological practice, particularly in complex diagnostic scenarios requiring individualized decision‐making. Prospective multicenter studies are warranted to externally validate this model and evaluate its applicability in diverse healthcare settings.

## 6 TAKE HOME MESSAGE

- A multivariable model combining PHI, PSA density, PSA l/t, PIRADS score, and age enables accurate identification of csPCa in biopsy‐naïve patients.
- The model achieved 100% sensitivity and safely avoided biopsies in nearly 50% of cases.
- The derived nomogram can support clinical decision‐making and outperform simplified strategies based on PHI or PIRADS alone.
- External validation of the Siddiqui et al. nomogram in our cohort further confirmed the robustness of PHI–mpMRI–based strategies across populations.

## AUTHOR CONTRIBUTIONS

*Conceptualization*: Mario Dominguez Esteban. *Methodology*: Mario Dominguez Esteban and Maria Teresa Garcia Unzueta. *Validation*: Jose Javier Gomez Roman, Alejandro Fernandez Florez and Maria Teresa Garcia Unzueta. *Formal analysis*: Mario Dominguez Esteban. *Investigation*: Ester Fernandez Guzman, Enrique Ramos Barselo, Ernesto Herrero Blanco, Sergio Zubillaga Guerrero, Roberto Ballestero Diego, Jaime Garcia Herrero, Marina Sanchez Gil, Guillermo Velilla Diez and Felix Campos Juanatey. *Resources*: Jose Javier Gomez Roman, Alejandro Fernandez Florez and Maria Teresa Garcia Unzueta. *Data curation*: Mario Dominguez Esteban. *Writing—original draft*: Mario Dominguez Esteban. *Writing—review and editing*: All authors. *Supervision*: Jose Luis Gutierrez Baños. All authors read and approved the final version of the manuscript.

## CONFLICT OF INTEREST STATEMENT

The authors declare no competing interests.

## REPORTING GUIDELINES

This study follows the STROBE and REMARK guidelines for observational and biomarker research.

## Supporting information


**Table S1:** Clinical, demographic, and pathological characteristics of the entire cohort (quantitative variables).
**Table S2:** Clinical, demographic, and pathological characteristics of the entire cohort (qualitative variables).
**Table S3:** Comparison of key clinical and pathological variables between patients with and without mpMRI.
