# Supplementary material for: Multivariable model integrating PHI and mpMRI for detecting csPCa in biopsy‐naïve men
Source: BJUI Compass. 2025 Dec 2;6(12):e70101. doi: 10.1002/bco2.70101 (PMC12672186; doi:10.1002/bco2.70101)
Supplement: Supplementary file 1 — Table S1: Clinical, demographic, and pathological characteristics of the entire cohort (quantitative variables). Table S2: Clinical, demographic, and pathological characteristics of the entire cohort (qualitative variables). Table S3: Comparison of key clinical and pathological variables between patients with and without mpMRI. [file BCO2-6-e70101-s001.docx]

**Appendix:**

Table-S1: *Clinical, demographic, and pathological characteristics of the entire cohort (quantitative variables)*

| **Variable** | **N** | **Mean** | **Median** | **SD** | **Minimum** | **Maximum** | **p Shapiro-Wilk** | **Normality** |
| --- | --- | --- | --- | --- | --- | --- | --- | --- |
| **Age** | 184 | 64.14 | 65.0 | 6.89 | 48.0 | 78.0 | 0.0078 | No |
| **Prostatic Volume** | 109 | 53.06 | 49.0 | 23.49 | 18.0 | 140.0 | < 0.0001 | No |
| **Pre-biopsy PSA** | 184 | 6.45 | 6.3 | 1.48 | 3.52 | 9.99 | 0.0009 | No |
| **Pre-biopsy Free PSA** | 179 | 1.05 | 0.9 | 0.86 | 0.2 | 8.0 | < 0.0001 | No |
| **Pre-biopsy PSA f/t Ratio** | 179 | 0.16 | 0.14 | 0.09 | 0.04 | 0.78 | < 0.0001 | No |
| **Pre-biopsy PSA Density** | 184 | 15.74 | 13.08 | 8.71 | 2.56 | 63.07 | < 0.0001 | No |
| **PHI (Prostate Health Index)** | 180 | 46.54 | 41.83 | 21.65 | 8.86 | 182.29 | < 0.0001 | No |
| **Prostatic Volume on TRUS (Biopsy)** | 184 | 52.97 | 45.0 | 33.15 | 15.0 | 300.0 | < 0.0001 | No |
| **Number of Cores Obtained** | 184 | 15.21 | 13.0 | 4.46 | 8.0 | 28.0 | < 0.0001 | No |
| **Number of Positive Cores** | 92 | 4.86 | 4.0 | 3.31 | 1.0 | 21.0 | < 0.0001 | No |
| **Affected Millimeters** | 92 | 23.5 | 19.5 | 22.84 | 1.0 | 156.0 | < 0.0001 | No |
| **Number of Negative Cores** | 100 | 10.73 | 9.0 | 5.46 | 2.0 | 28.0 | < 0.0001 | No |
| **Number of Cores with Higher Gleason Grade** | 92 | 3.27 | 2.0 | 2.56 | 1.0 | 10.0 | < 0.0001 | No |
| **Affected Millimeters with Higher Gleason Grade** | 92 | 16.47 | 10.0 | 16.58 | 1.0 | 93.0 | < 0.0001 | No |
| **Number of Cores with Lower Gleason Grade** | 39 | 3.31 | 3.0 | 2.7 | 1.0 | 14.0 | < 0.0001 | No |
| **Affected Millimeters with Lower Gleason Grade** | 39 | 14.17 | 9.0 | 19.15 | 0.5 | 117.0 | < 0.0001 | No |

Table-S2: *Clinical, demographic, and pathological characteristics of the entire cohort (qualitative variables):*

| **Variable** | **Label** | **Frequency** | **Percentage (%)** |
| --- | --- | --- | --- |
| **PSA f/t Ratio (<20 or ≥20)** | <20 | 136 | 73.91 |
|  | ≥20 | 43 | 23.37 |
|  | Unknown | 5 | 2.72 |
| **PSA f/t Ratio (<15 or ≥15)** | <15 | 108 | 58.7 |
|  | ≥15 | 71 | 38.59 |
|  | Unknown | 5 | 2.72 |
| **PSA Density (<20 or ≥20)** | <20 | 130 | 70.65 |
|  | ≥20 | 54 | 29.35 |
| **Digital Rectal Exam (Volume)** | II | 115 | 62.5 |
|  | III | 32 | 17.39 |
|  | I | 27 | 14.67 |
|  | Unknown | 7 | 3.8 |
|  | IV | 3 | 1.63 |
| **Digital Rectal Exam (Stages I-II vs III-IV)** | I-II | 142 | 77.17 |
|  | III-IV | 35 | 19.02 |
|  | Unknown | 7 | 3.8 |
| **Suspicious Digital Rectal Exam (DRE)** | No | 140 | 76.09 |
|  | Yes | 37 | 20.11 |
|  | N/A | 7 | 3.8 |
| **Biopsy Indication** | High PSA | 147 | 79.89 |
|  | Both | 36 | 19.57 |
|  | Positive DRE | 1 | 0.54 |
| **Biopsy Result** | Positive | 92 | 50.0 |
|  | Negative Result | 92 | 50.0 |
| **Clinically Significant Cancer Result** | Negative Result | 92 | 50.0 |
|  | No | 55 | 29.89 |
|  | Yes | 37 | 20.11 |
| **Candidate for Active Surveillance** | Negative Result | 92 | 50.0 |
|  | No | 74 | 40.22 |
|  | Yes | 18 | 9.78 |
| **Highest PIRADS on MRI** | No RMN | 97 | 52.72 |
|  | 4 | 41 | 22.28 |
|  | 2 | 21 | 11.41 |
|  | 3 | 16 | 8.7 |
|  | 5 | 6 | 3.26 |
|  | 1 | 3 | 1.63 |
| **PIRADS Score on MRI (1–2 vs 3–4–5)** | No MRI | 97 | 52.72 |
|  | [3-4-5] | 63 | 34,24 |
|  | [1-2] | 24 | 13.04 |
| **Location of Highest PIRADS Lesion** | Unknown or No MRI | 99 | 53.8 |
|  | Bilateral | 24 | 13.04 |
|  | Apex | 17 | 9.24 |
|  | Left Prostatic Lobe | 14 | 7.61 |
|  | Central Gland | 13 | 7.07 |
|  | Central and Peripheral Gland | 10 | 5.43 |
|  | Right Prostatic Lobe | 7 | 3.8 |
| **Suspicious TRUS Finding (During Biopsy)** | No | 156 | 84.78 |
|  | Sí | 28 | 15.22 |
| **Location of Suspicious TRUS Finding (Biopsy)** | Unknown | 155 | 84.24 |
|  | Left Prostatic Lobe | 11 | 5.98 |
|  | Ápex | 6 | 3.26 |
|  | Bilateral | 5 | 2.72 |
|  | Right Prostatic Lobe | 5 | 2.72 |
|  | No lesion | 2 | 1.09 |
| **Highest Gleason Score after Biopsy** | Negative Result | 92 | 50.0 |
|  | 3+3 | 45 | 24.46 |
|  | 3+4 | 27 | 14.67 |
|  | 4+4 | 12 | 6.52 |
|  | 4+3 | 4 | 2.17 |
|  | 4+5 | 2 | 1.09 |
|  | 5+5 | 1 | 0.54 |
|  | 5+4 | 1 | 0.54 |
| **Location of Highest Gleason after Biopsy** | Negative Result | 92 | 50.0 |
|  | Left Prostatic Lobe | 34 | 18.48 |
|  | Bilateral | 26 | 14.13 |
|  | Right Prostatic Lobe | 23 | 12.5 |
|  | Ápex | 7 | 3.8 |
|  | Central Gland | 2 | 1.09 |

Table S3: **Comparison of key clinical and pathological variables between patients with and without mpMRI.**

| **Quantitative Variables *** | | **No MRI (n = 97)** | | **MRI (n = 87)** | | | **p-value** | |
| --- | --- | --- | --- | --- | --- | --- | --- | --- |
|  | | **Median** | **RIQ** | **Median** | | **RIQ** |  | |
| **Age (years)** | | 65 | 12 | 64 | | 9.5 | 0.814 | |
| **Pre-biopsy PSA** | | 6.33 | 2.35 | 6.27 | | 2.25 | 0.387 | |
| **Pre-biopsy Free PSA** | | 0.9 | 0.63 | 0.8 | | 0.6 | 0.2773 | |
| **Pre-biopsy PSA f/t Ratio** | | 0.14 | 0.09 | 0.12 | | 0.08 | 0.1814 | |
| **Pre-biopsy PSA Density** | | 13.12 | 10.77 | 13.04 | | 11.19 | 0.8635 | |
| **Prostate Health Index (PHI)** | | 39.74 | 23.8 | 42.31 | | 22.91 | 0.2344 | |
| **Prostatic Volume (cc)** | | 45 | 34 | 43 | | 39 | 0.9834 | |
| **Number of Cores Obtained** | | 12 | 3 | 15 | | 8 | **< 0.001** | |
| **Number of Positive Cores** | | 4 | 5 | 5 | | 4.25 | 0.1625 | |
| **Affected Millimeters (mm)** | | 17 | 20.5 | 22 | | 22.5 | 0.1569 | |
| **Number of Negative Cores** | | 9 | 5 | 9 | | 7 | 0.2445 | |
| **Number of Cores with Higher Gleason Grade** | | 2 | 3.25 | 2.5 | | 4 | 0.7729 | |
| **Affected Millimeters with Higher Gleason Grade** | | 10 | 15.25 | 9.5 | | 22 | 0.3935 | |
| **Number of Cores with Lower Gleason Grade** | | 2 | 2.5 | 3 | | 2.5 | 0.4054 | |
| **Affected Millimeters with Lower Gleason Grade (mm)** | | 9 | 11.5 | 9 | | 13.5 | 0.9543 | |
|  | |  | | | | | | |
| **Qualitative Variables**** | **No MRI (n = 97)** | | | **MRI (n = 87)** | | |  | **p** |
|  |  | | **n** | **%** | **n** | | **%** |  |
| **DRE** | I-II | | 75 | 79.8 | 67 | | 80.7 | 0.999 |
|  | III - IV | | 19 | 20.2 | 16 | | 19.3 |  |
| **Suspicious DRE** | No | | 75 | 79.8 | 68 | | 78.3 | 0.956 |
|  | Yes | | 19 | 20.2 | 18 | | 21.7 |  |
| **Biopsy Indication** | High PSA | | 78 | 80.4 | 69 | | 79.3 | 0.712 |
|  | DRE positive | | 1 | 1.0 | 0 | | 0.0 |  |
|  | Both | | 18 | 18.6 | 18 | | 20.7 |  |
| **Biopsy Result** | Negative | | 53 | 54.6 | 39 | | 44.8 | 0.237 |
|  | Positive | | 44 | 45.4 | 48 | | 55.2 |  |
| **Highest Gleason Score after Biopsy** | 3+3 | | 23 | 52.3 | 22 | | 45.8 | 0.520 |
|  | 3+4 | | 12 | 27.3 | 15 | | 31.2 |  |
|  | 4+3 | | 6 | 13.6 | 6 | | 12.5 |  |
|  | 4+4 | | 1 | 2.3 | 2 | | 4.2 |  |
|  | 5+3 | | 1 | 2.3 | 0 | | 0.0 |  |
|  | 5+4 | | 1 | 2.3 | 0 | | 0.0 |  |
| **Lower Gleason Grade after Biopsy** | 3+3 | | 13 | 81.2 | 21 | | 91.3 | 0.441 |
|  | 3+4 | | 1 | 6.2 | 1 | | 4.3 |  |
|  | 4+3 | | 1 | 6.2 | 0 | | 0.0 |  |
|  | 4+4 | | 1 | 6.2 | 1 | | 4.3 |  |
|  | 5+4 | | 0 | 0.0 | 1 | | 4.3 |  |
| **Prostate Cancer Risk** | Very Low | | 10 | 22.7 | 8 | | 16.7 | 0.840 |
|  | Low | | 13 | 29.5 | 14 | | 29.2 |  |
|  | Intermedium-Low | | 12 | 27.3 | 11 | | 22.9 |  |
|  | Intermedium-High | | 4 | 9.1 | 6 | | 12.5 |  |
|  | High | | 8 | 18.2 | 8 | | 16.7 |  |
| **Clinically Significant Cancer Result** | No | | 23 | 52.3 | 22 | | 45.8 | 0.683 |
|  | Yes | | 21 | 47.7 | 26 | | 54.2 |  |
| **Candidate for Active Surveillance** | No | | 35 | 79.5 | 39 | | 81.2 | 0.999 |
|  | Sí | | 9 | 20.5 | 9 | | 18.8 |  |

* Results are presented as median and interquartile range (IQR) in the **quantitative variables**. Group comparisons were performed using the non-parametric Mann–Whitney U test (MW).

** Comparison of the main characteristics of qualitative variables in patients with/without MRI. χ² test
